# Optimal cut‐off points of fat mass index and visceral adiposity index associated with type 2 diabetes mellitus

**DOI:** 10.1002/fsn3.2874

**Published:** 2022-04-18

**Authors:** Amir Bagheri, Tina Khosravy, Mehdi Moradinazar, Seyed Mostafa Nachvak, Yahya Pasdar, Davood Soleimani, Mehnoosh Samadi

**Affiliations:** ^1^ 48439 Department of Community Nutrition School of Nutritional Sciences and Dietetics Tehran University of Medical Sciences Tehran Iran; ^2^ 48468 Department of Health Nutrition Lorestan University of Medical Sciences Lorestan Iran; ^3^ 48464 Department of Nutritional Sciences School of Nutritional Sciences and Food Technology Kermanshah University of Medical Sciences Kermanshah Iran; ^4^ 48464 Research Center for Environmental Determinants of Health (RCEDH) School of Public Health Kermanshah University of Medical Sciences Kermanshah Iran; ^5^ 48464 Research Center of Oils and Fats Kermanshah University of Medical Sciences Kermanshah Iran

**Keywords:** fat mass index, obesity, type 2 diabetes, visceral adiposity index

## Abstract

**Background:**

Indices, based on anthropometrics with or without non‐anthropometric components, are predictive of cardio‐metabolic outcomes. Fat mass index (FMI) is similar to BMI except measured fat mass replaces body weight. The visceral adiposity index (VAI) combines anthropometric measures with lipid measurements TG/HDL. The relationship of these indices to incident type 2 diabetes (T2DM) has not been established. Therefore, we have evaluated the predictive power and optimal cut‐off points of FMI, and VAI with T2DM in a cross‐sectional population study.

**Methods:**

These population‐based cross‐sectional study comprised 8411 adults aged 35–65 years using data from the Ravansar Non‐Communicable Diseases (RaNCD) cohort. VAI and FMI were defined as previously published. Optimal cut‐off points for association with incident T2DM were determined from receiver‐operating curves (ROC).

**Results:**

The optimal cut‐off point for VAI was 4.86 (AUC: 0.673; 95% CI: 0.65–0.69) and FMI 9.3 (AUC: 0.57; 95% CI: 0.55–0.59), and for T2DM in our study population. The odds ratios (OR) for T2DM were nearly identical, for VAI 1.098 (95% CI: 1.08–1.11) and for FMI 1.08 (95% CI: 1.05–1.10).

**Conclusions:**

In the current population study, VAI and FMI were weakly associated with T2DM. Therefore, it seems that anthropometric measures are unlikely to be strong mediators of T2DM compared to historical and other factors in the population studied.

## Abstract 2

We found that VAI had more predictive power to diagnose T2DM than FMI.

## 1 INTRODUCTION

Obesity is associated with type 2 diabetes (T2DM), and plays an important role in the development of its side effects (Chobot et al., 2018). T2DM is a major global health threat and the incident is rapidly increasing in Asian population (Zheng et al., 2018). In Iran, about 10,000 people die due to diabetes every year, and emerging prevalence of both diabetes and obesity is a major health challenge for health care professionals in the forthcoming years (Veisani et al., 2018).

The body mass index (BMI) is a measure of body size and is commonly used as to classify obesity and overweight, and as a predictor of obesity‐related diseases. However, it has several limitations (Meigs et al., 2006); first, it does not distinguish differences in body composition such as between total body fat and lean mass (Nevill et al., 2006). Second, it does not reflect differences in body fat distribution and body shape (González et al., 2007; Nevill et al., 2006). Waist circumference (WC) has been used to diagnose abdominal obesity but has a high correlation with BMI (Krakauer & Krakauer, 2012; Pouliot et al., 1994). Therefore, new anthropometric based indices, including fat mass index (FMI) and visceral adiposity index (VAI), have been suggested to better reflect abdominal obesity. VAI was derived as a mathematic model based on WC, BMI, triglyceride (TG), and high‐density lipoprotein (HDL). Studies have shown that VAI reflects the presence of visceral fat (Amato & Giordano, 2014; Amato et al., 2010). This index is felt to be reliable marker of visceral fat induced metabolic dysfunction and is associated with cardio‐metabolic risk (Amato et al., 2010) and metabolic syndrome (Elisha et al., 2013). FMI is expressed as body fat mass divided by height^2^, and seems to identify risk better than percent body fat (VanItallie et al., 1990). Studies showed that FMI is an independent risk factor for metabolic syndrome and cardiovascular disease (Liu et al., 2013).

Some studies have suggested that VAI predicts T2DM (Gu et al., 2018; Liu et al., 2016). However, other studies have not been confirmatory (Bozorgmanesh et al., 2011; Wang et al., 2015). Furthermore, to our knowledge, there is no study to investigate the optimal cut‐off point of FMI and its relationship with T2DM. Importantly, earlier studies identified some differences in cut‐off points across populations (Nusrianto et al., 2019). Therefore, the current study was conducted to survey the association of FMI and VAI with T2DM and to detect the optimal cut‐off points with incident T2DM, in the Kurdish population residents in the West of Iran.

## 2 MATERIALS AND METHODS

### 2.1 Study design and subjects

The current population‐based cross‐sectional study was conducted on the baseline data obtained from the Ravansar cohort study (RaNCD). The RaNCD study is a subdivision of a prospective epidemiological research study in Iran (PERSIAN), and is the first cohort study in the Kurdish population. Ravansar is a city with about 50,000 people, mainly from Iranian Kurdish ethnicity, located in the west of Iran in the Kermanshah province. About 10,000 permanent residents of Ravansar in the age range of 35–65 (both men and women) were included in this cohort. Additional details of the RaNCD study protocol have previously been published (Hamzeh et al., 2020; Pasdar et al., 2019; Poustchi et al., 2018).

### 2.2 Inclusion and exclusion criteria

In total, subjects with the following features were excluded from the RaNCD cohort study: those who live in Ravansar lower than 9 months a year; participants who did not want to attend the study; subjects that are new inhabitants (under 1 year), and people who cannot participate in the cohort study because of mental or physical disability or any acute psychological disorder, blindness, deafness, and dumbness.

For the current study, people with incomplete and missing information, those with diabetes, CVD, cancer, and thyroid diseases at the beginning of the study, pregnant women, those whose calorie‐restricted diets, or consumption of hormonal drugs might have affected body weight were excluded.

Therefore, participants without the aforementioned criteria were included.

### 2.3 Data collection

The general characteristics of individuals, including age, sex, marital status, income, number of years education, place of residence (urban, rural), age at onset of diabetes in the patient group, history of chronic disease, and tobacco and alcohol consumption, were collected by trained interviewers and registered in the electronic online data form.

To evaluate the physical activity level of participants, a physical activity questionnaire standardized in Persian Cohort was used. The questionnaire comprised 22 questions about the individual's daily activities. Based on activity intensity, physical activity was expressed as light, moderate, and high.

### 2.4 Anthropometry Measurements

Based on the Persian cohort protocol, body weight and height were measured with body 770 (Inbody Co) and BSM 370 (Biospace Co), respectively. The precision of the measurements for weight was 0.5 kg, and for height it was 0.1 cm. WC was measured in the narrowest area, the distance between below the last rib and above the iliac spine in the exhaled state. Other components of body composition, including percentage of body fat (PBF), body fat mass (BFM), and fat free mass (FFM), were measured with Inbody 770 (Inbody Co). Weight (kg) divided by height squared (m^2^) was employed to calculate body mass index (BMI). Finally, VAI (Amato & Giordano, 2014) and FMI (Liu et al., 2013) were calculated by the following formula:

$$\text{VAI}=\text{Males}:\left(\frac{\text{WC}}{39.68+\left(1.88\times \text{BMI}\right)}\right)\times \left(\frac{\text{TG}}{1.03}\right)\times \left(\frac{1.31}{\text{HDL}}\right)$$

$$\text{VAI}=\text{Females}:\left(\frac{\text{WC}}{39.58+\left(1.89\times \text{BMI}\right)}\right)\times \left(\frac{\text{TG}}{0.81}\right)\times \left(\frac{1.52}{\text{HDL}}\right)$$

$$\text{FMI}=\text{BFM}/\text{height}{\left(m\right)}^{2}$$

### 2.5 Biochemical factors

Blood sample assessment was detailed in the study protocol. Briefly, fasting blood samples were collected from the ante‐brachial vein. Then, they were centrifuged and stored in aliquots in cryotubes at −80°C until further analyzed. Commercially available kits were utilized to measure serum levels of FBS and lipid profiles.

### 2.6 Type 2 diabetes and blood pressure

T2DM was defined if the subject's FBS level was ≥126 mg/dl or they took glucose‐lowering medications. After resting for 10 min in a sitting position, blood pressure was measured from both right and left arms for two times from each side with an interval of 5 min, by a manometer (Riester) cuff and stethoscope (Riester). Then, the mean of final systolic and diastolic blood pressure was calculated.

### 2.7 Statistical analyses

All information was entered into STATA 14 software for analysis. To examine the relationship between qualitative variables (sex, marital status, economic status, educational status, smoking, and physical activity) with T2DM, Chi‐square test was run. One‐way analysis of variance (ANOVA) was performed to evaluate the association between quantitative variables (age, BMI, and total energy) with T2DM. Pearson's correlation coefficient was used to assess the correlation between the anthropometric indices and Bonferroni adjustment was used to calculate significance levels. To investigate the association of VAI and FMI with T2DM, logistic regression model was conducted in the general population and for gender subgroup. Along with the crude model, three other models were adopted to eliminate the effect of potential confounders. In model 1, age, sex, marital status, and education level were adjusted as confounding variables. Model 2 was further adjusted for socioeconomic status, smoking, alcohol consumption, medication use, and physical activity. Model 3 was adjusted for blood pressure and total caloric intake as well. ROC analysis was also employed to estimate and compare area under the ROC curve (AUC), sensitivity, and specificity of the anthropometric indices, and to determine the optimal cut‐off point for each of them in relation to type 2 diabetes. *p*‐value of < .05 was considered as significant.

## 3 RESULTS

Out of 10,000 participants in the Ravansar cohort, 8411 met the inclusion criteria. General characteristics of participants are shown in Table 1. Out of 8411 included subjects, 553 (6.3%) had T2DM and 7858 (93.6%) were healthy. There was a significant difference between healthy and diabetic groups in age, marital status, level of education, place of residence (urban, rural), smoking, body mass index, blood pressure, and physical activity.

**TABLE 1:** Demographic characteristics of the included participants

Variables	Stratification	Healthy *N* (%)	T2DM *N* (%)	*p*‐value^a^
Health condition	All	7858 (93.4)	553 (6.6)	^_^
Gender	Men	3998 (93)	298 (7)	.171
	Women	3860 (93.8)	255 (6.2)	
Age (year)	35–45	3879 (66.8)	126 (3.2)	.001>
	45–55	2557 (91.5)	235 (8.5)	
	55–65	1422 (88.1)	192 (11.9)	
Marital status	Married	7072 (93.2)	515 (6.8)	.01
	Single	786 (95.4)	38 (4.6)	
Education level (year)	Illiterate	1734 (92.1)	149 (7.9)	.002
	≤5 years	2969 (93.1)	221 (6.9)	
	6–9 years	1387 (93.1)	95 (6.4)	
	10–12 years	1090 (95.5)	51 (4.5)	
	≤13 years	678 (94.8)	37 (5.2)	
Economic status	1	1541 (94.2)	95 (5.8)	.598
	2	1549 (92.8)	120 (7.2)	
	3	1549 (93.2)	112 (6.8)	
	4	1578 (93.5)	102 (6.5)	
	5	1602 (93.6)	110 (6.4)	
Place of Residency	Urban	4657 (92.8)	363 (7.2)	.003
	Rural	3201 (94.4)	190 (5.6)	
Socioeconomic status	1	1556 (93.8)	103 (6.2)	.391
	2	1541 (94.1)	96 (5.9)	
	3	1570 (92.3)	124 (7.3)	
	4	1562 (92.9)	119 (7.1)	
	5	1628 (93.7)	110 (6.3)	
Alcohol consumption	NO	7322 (93.4)	516 (6.6)	.906
	YES	536 (93.5)	37 (6.5)	
Smoking	NO	990 (93.7)	67 (6.3)	<.001
	YES	598 (88.9)	75 (11.1)	
BMI	19–24.9	2337 (95.9)	100 (4.1)	<.001
	25–29.9	3392 (92.9)	261 (7.14)	
	30–34.9	1533 (91.5)	143 (8.5)	
	>35	402 (91.2)	39 (8.8)	
Physical activity	low	2070 (26.3)	173 (31.2)	.003
	Moderate	3971 (50.5)	283 (51.1)	
	high	1813 (23.08)	97 (17.5)	

Note

Data are reported as Frequency (percentage).

Abbreviations: BMI, Body Mass Index; T2DM, Type 2 Diabetes Mellitus.

^a^ *p*‐value was calculated using Chi‐square tests.

Table 2 shows the mean and SD of VAI and FMI for the total study population as well as healthy and diabetes men and women. FMI and VAI were significantly higher in total, men, and women affected by T2DM than in healthy populations (*p* < .001). Table 3 shows correlation coefficients between anthropometric indices across all population. The highest significant positive correlation was observed for FMI and BMI (*r* = 0.91, *p* < .001).

**TABLE 2:** Mean and SD of the anthropometric indices in healthy and diabetic individuals

Variables		Healthy	T2DM	*p*‐value^a^
		Mean ± SD	Mean ± SD	
FMI (kg/m^2^)	All	9.2 ± 3.9	10.2 ± 3.7	<.001
	Men	7.1 ± 2.8	8.4 ± 2.9	<.001
	Women	11.5 ± 3.7	12.3 ± 3.4	<.001
VAI	All	5.1 ± 3.8	8.1 ± 8.0	<.001
	Men	4.9 ± 3.7	7.4 ± 6.3	<.001
	Women	5.3 ± 3.9	8.9 ± 9.5	<.001

Notes

Data are reported as mean and standard deviation.

Abbreviations: FMI, Fat Mass Index; T2DM, Type 2 Diabetes Mellitus; VAI, Visceral Adiposity Index.

^a^ *p*‐value was calculated using the independent samples *t*‐test.

**TABLE 3:** Correlation coefficient between the anthropometric indices in all population

Variables	Gender	FMI	VAI	BMI
FMI	Both	‐‐‐‐		
	Men	‐‐‐‐		
	Women	‐‐‐‐		
VAI	Both	0.03^**^	‐‐‐‐	
	Men	0.204^***^	‐‐‐‐	
	Women	0.121^***^	‐‐‐‐	
BMI	Both	0.91^***^	0.13^***^	‐‐‐‐
	Men	0.928^***^	0.246^***^	‐‐‐‐
	Women	0.969^***^	0.142^***^	‐‐‐‐
WC	Both	0.716^***^	0.186^***^	0.807^***^
	Men	0.774^***^	0.237^***^	0.796^***^
	Women	0.806^***^	0.178^***^	0.819^***^

Abbreviations: BMI, Body Mass Index; FMI, Fat Mass Index; VAI, Visceral Adiposity Index.

**p < .01, ***p < .001.

**FIGURE 1:**
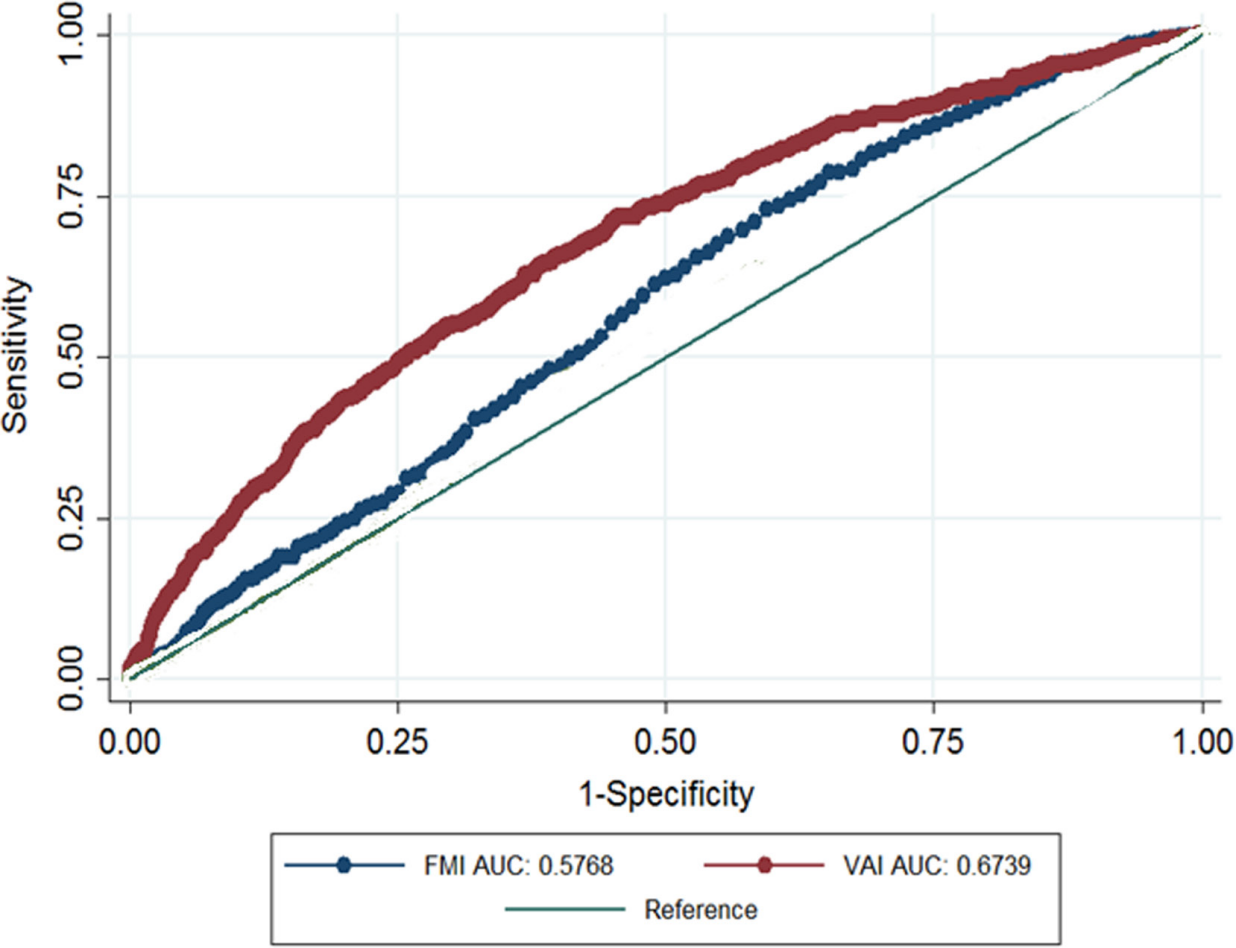
AUC of FMI and VAI versus reference line in relation to type 2 diabetes in general populations

**FIGURE 2:**
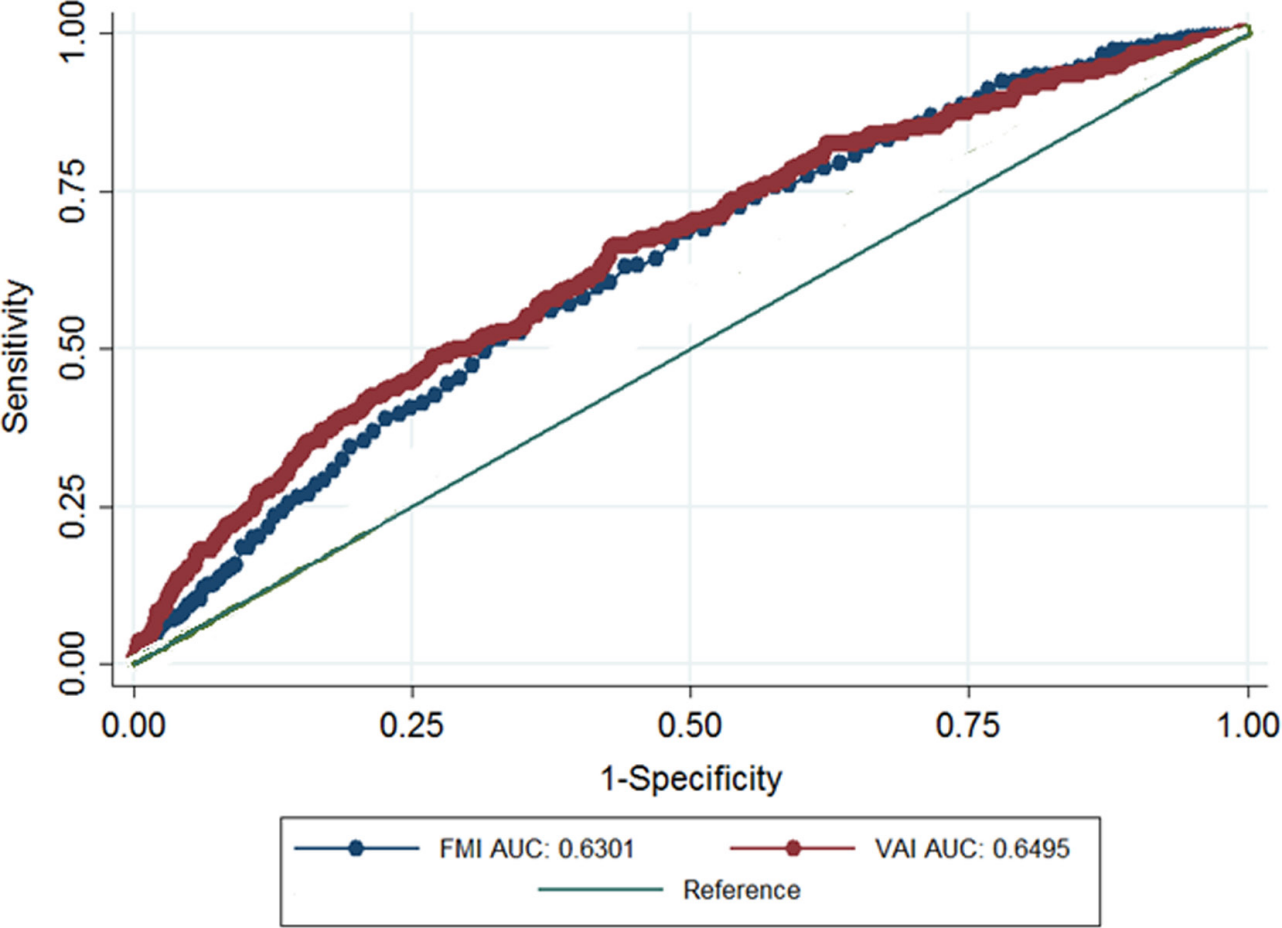
AUC of FMI and VAI versus reference line in relation to type 2 diabetes in men

**FIGURE 3:**
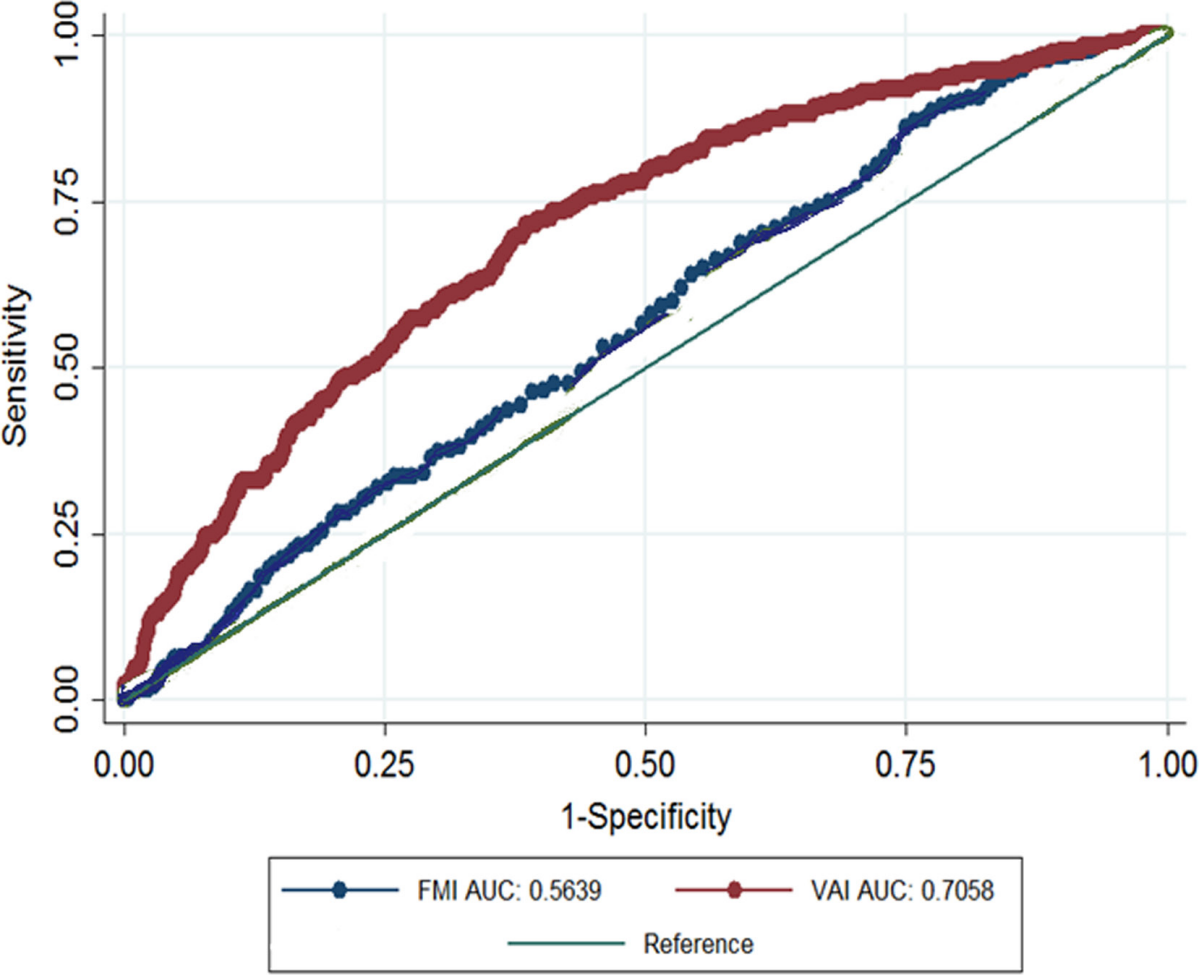
AUC of FMI and VAI versus reference line in relation to type 2 diabetes in women

Sensitivity, Specificity, and Area under the ROC Curve of two indices for identify T2DM are shown in Table 4. The optimal cut‐off points for VAI were 4.85 (sensitivity 65.75, specificity 60.18) in the general population, 4.82 (sensitivity 60.15, specificity 61.07) in men, and 5.1 (sensitivity 71.37, specificity 61.55) in women. The optimal cut‐off point for FMI was 9.3 in the total population, 7.5 in men, and 11.7 in women. In the general population, ROC analysis showed that VAI had the greatest association with T2DM (AUC: 0.67; 95%CI: 0.65–0.69) than FMI. Moreover, in men and women, VAI had more association with T2DM (Table 4) (Figures 1, 2, 3).

**TABLE 4:** AUC and optimal cut‐off points* of the anthropometric indices in relation to type 2 diabetes

Variables		AUC (% 95 CI)	Cut‐off point	Sensitivity	specificity	Positive predictive value	Negative predictive value	Likelihood ratio (+)	Likelihood ratio (−)
FMI	All	0.576 (0.55–0.59)	≤9.3	56.41	54.0	54.3	55.7	1.28	0.72
	Men	0.630 (0.59–0.66)	≤7.5	60.47	57.33	58.2	59.7	1.37	0.70
	Women	0.563 (0.52–0.59)	≤11.7	60.33	53.97	56.0	57.1	1.26	0.75
VAI	All	0.673 (0.65–0.69)	≤4.859	65.75	60.18	61.9	63.1	1.65	0.58
	Men	0.649 (0.61–0.68)	≤4.821	60.15	61.07	60.6	60.4	1.31	0.74
	Women	0.705 (0.67–0.73)	≤5.106	71.37	61.55	64.5	68.7	1.53	0.64

Abbreviations: AUC, Area Under the Curve; FMI, Fat Mass Index; T2DM, Type 2 Diabetes Mellitus; VAI, Visceral Adiposity Index.

Results from multivariable logistic regression analysis indicated the association of VAI and FMI and odds of T2DM as illustrated in Table 5. After adjustment for all potential confounders, subjects above the cut‐off point for VAI had higher odds of T2DM (OR: 1.98; 95% CI: 1.08–1.11) than those below the cut‐off point. Stratified for gender indicated that men and women in higher cut‐off points had greater odds of T2DM (OR: 1.09; 95% CI: 1.07–1.12) and (OR: 1.10; 95% CI: 1.07–1.12), respectively. Moreover, participants above the cut‐off point for FMI had a greater likelihood of T2DM (OR: 1.08; 95% CI: 1.05–1.10). After stratification for gender, the odds of T2DM for men and women in higher cut‐off points of this index were OR: 1.13; 95% CI: 1.09–1.18 and OR: 1.04; 95% CI: 1.01–1.08, respectively.

**TABLE 5:** Odds ratio and 95% confidence interval of different anthropometric indices with type 2 diabetes

		Model 0	Model 1	Model 2	Model 3
		OR (% 95 CI)			
FMI	All	1.06 (1.03–1.08)	1.09 (1.06–1.12)	1.08 (1.06–1.11)	1.08 (1.05–1.10)
	Men	1.15 (1.11–1.20)	1.15 (1.11–1.19)	1.14 (1.10–1.19)	1.13 (1.09–1.18)
	Women	1.05 (1.02–1.09)	1.05 (1.01–1.08)	1.05 (1.01–1.08)	1.04 (1.01–1.08)
VAI	All	1.10 (1.08–1.12)	1.10 (1.08–1.11)	1.10 (1.08–1.11)	1.098 (1.08–1.11)
	Men	1.09 (1.07–1.12)	1.10 (1.08–1.12)	1.10 (1.07–1.12)	1.09 (1.07–1.12)
	Women	1.10 (1.08–1.13)	1.10 (1.07–1.12)	1.10 (1.07–1.12)	1.10 (1.07–1.12)

Note

Model 0: Crude model.

Model 1: Adjusted for age, sex, marital status, and level of education.

Model 2: Further adjusted for socioeconomic status, smoking, drug and alcohol consumption, and physical activity.

Model 3: Additional adjusted for blood pressure and total caloric intake.

Abbreviations: CI, Confidence Interval; FMI, Fat Mass Index; OR, Odds Ratio; T2DM, Type 2 Diabetes Mellitus;VAI, Visceral Adiposity Index.

## 4 DISCUSSION

Our findings indicated that VAI had the highest AUC for incident T2DM followed by FMI. It means that VAI is more associated with T2DM than another index. Moreover, multivariable logistic regression showed that subjects in high cut‐off points of VAI and FMI had significantly greater odds of having T2DM.

In the current study, the optimal cut‐off point of VAI was 4.85 in the general population, 4.82 in men, and 5.2 in women. Furthermore, we found that higher VAI scores were associated with higher odds of T2DM. In line with our findings, two studies conducted in Iran showed a significant association between an increase in VAI and T2DM risk (Bozorgmanesh et al., 2011; Janghorbani & Amini, 2016). Also, one of them showed that VAI was a strong predictor of T2DM, but their predictive power was almost the same as WHR, BMI, and WC (Janghorbani & Amini, 2016). Cross‐sectional studies conducted in China revealed that a higher VAI score was associated with T2DM (Gu et al., 2018; Liu et al., 2016). Three prospective cohort studies in China also clarified that VAI was an independent predictor for T2DM incidence (Chen et al., 2014; Wang et al., 2015; Zhang et al., 2016). Due to the proposed association between VAI and visceral adipose tissue in the development of insulin resistance (Amato et al., 2010; Ciresi et al., 2017), T2DM (Janghorbani & Amini, 2016), inflammation (Amato & Giordano, 2014, Amato et al., 2014), and metabolic syndrome (Amato et al., 2010), suggest that VAI was an assessment tool for T2DM.

We showed that the optimal cut‐off point of FMI to predict incident T2DM was 9.3 in the total population, 7.5 in men, and 11.7 in women. Moreover, this index was significantly associated with increased odds of incident T2DM. To our knowledge, there is no other studies of FMI cut‐off points and T2DM. A cross‐sectional study of 1698 adults aged 20–79 years in China indicated that subjects in the highest quartile of FMI had greater odds of metabolic syndrome (Liu et al., 2013). They also revealed that the optimal cut‐off point of FMI was 7.00 kg/m^2^ for men and 7.90 kg/m^2^ for women which could effectively predict metabolic syndrome. It seems that more studies are needed in this area.

The large sample size was a major strength of the current study. We provided, for the first time, the optimal cut‐off point for FMI in relation to T2DM. Also, the anthropometric and laboratory data were collected as part of a comprehensive study prospective (RaNCD). Nevertheless, there are some limitations. First, the cross‐sectional design cannot establish causal associations. Second, we could not investigate the association of these indices with hemoglobin A1c, fasting insulin, and insulin resistance. Finally, it must be noted that this study was carried out on Kurdish adults who live in the west part of Iran, so the results to other ethnicity and age groups must be generalized with caution.

## 5 CONCLUSION

VAI had more predictive ability for the presence of T2DM than FMI. We found that for incident T2DM in the Kurdish population, the optimal cut‐off point for VAI was 4.85 and for FMI 9.3. Prospective cohort studies are needed to evaluate the risk of future diabetes from baseline measurement of these and other indices.

## CONFLICT OF INTEREST

All authors declare that they have no conflicts of interests.

## ETHICAL APPROVAL

This study was approved by the Ethics Committee of Kermanshah University of Medical Sciences (IR.KUMS.REC.1398.158).

## CONSENT TO PARTICIPATE

First, the aims of the study were clarified to the participants and then all participants were requested to complete a written informed consent before data collection. This study was conducted in accordance with the declaration of Helsinki.

## CONSENT FOR PUBLICATION

All authors approved the manuscript for submission.

## Data Availability

The raw data supporting the conclusions of this article will be made available by the authors, on reasonable request to the corresponding author.
